# First person – Namrata Kulkarni

**DOI:** 10.1242/dmm.053075

**Published:** 2026-07-24

## Abstract

First Person is a series of interviews with the first authors of a selection of papers published in Disease Models & Mechanisms, helping researchers promote themselves alongside their papers. Namrata Kulkarni is first author on ‘ Fos regulates age-dependent neuroinflammation in a *VAP^P58S^* model of amyotrophic lateral sclerosis’, published in DMM. Namrata conducted the research described in this article while a PhD research scholar in Professor Girish Ratnaparkhi's lab at Indian Institute of Science Education and Research (IISER) Pune, Pune, India. She is now a Scientist – Skin Biology and Product Efficacy in the lab of Dr Deeleep Rout at Almora Botanica India Private Limited, Bangalore, India, investigating the molecular mechanisms of ageing and neurodegeneration to identify novel therapeutic targets for translational medicine.

**Figure:**
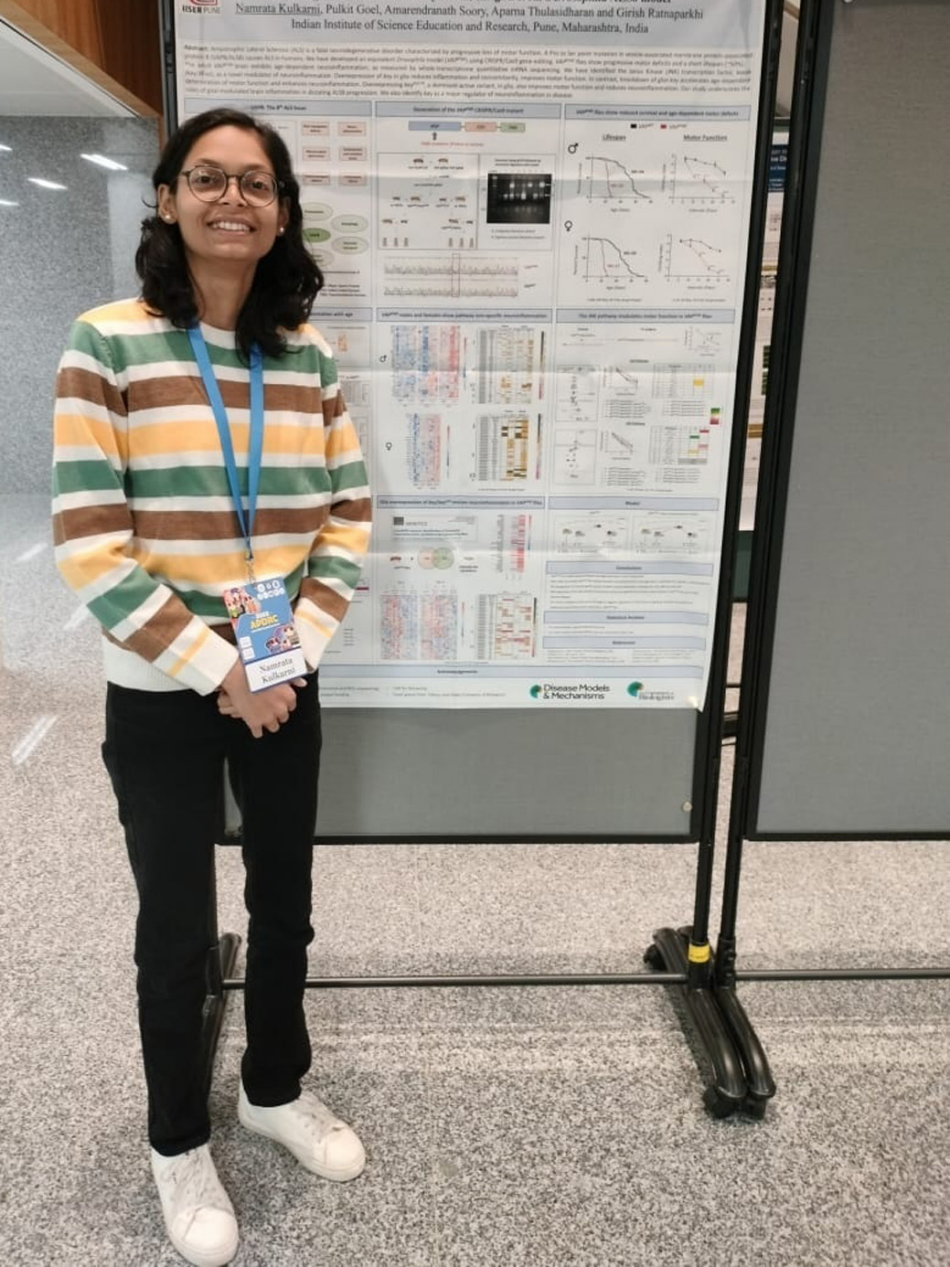
Namrata Kulkarni

**Who or what inspired you to become a scientist?**

I have always been fascinated by the process of scientific discovery and its potential to improve human health. My interest in neurodegenerative diseases began during my Master's research, when I was first exposed to the complexity of neuronal dysfunction and degeneration. This interest grew further during my PhD, when I worked on amyotrophic lateral sclerosis (ALS) and explored mechanisms underlying disease progression. I was particularly drawn to the fact that, despite significant advances in biomedical research, many neurodegenerative disorders still lack effective treatments. The complexity of the nervous system and the unanswered questions surrounding these diseases continue to motivate me as a scientist. Through my research, I hope to contribute to a better understanding of disease mechanisms and, ultimately, to the development of more effective therapeutic strategies for patients.

**What is the main question or challenge in disease biology you are addressing in this paper? How did you go about investigating your question or challenge?**

ALS is a complex neurodegenerative disease caused by mutations in multiple genes, with *VAPB* being associated with ALS8. In this study, we sought to understand how expression of the ALS-associated *VAP^P58S^* mutation affects disease progression and motor function using a *Drosophila* model. We found that mutant flies exhibited a shortened lifespan and progressive age-dependent motor impairments that closely resembled key features of the disease. Importantly, these defects were accompanied by an increase in neuroinflammation as the animals aged. We then investigated whether modulating inflammatory pathways could alter disease outcomes and found that glial overexpression of Fos reduced inflammation and significantly improved motor function in the mutant flies. Our findings highlight the contribution of glial-mediated inflammation to ALS pathology and suggest that targeting inflammatory pathways may offer a potential therapeutic strategy.

Our findings suggest that excessive inflammation may contribute to ALS progression and that targeting it could help slow the disease in the future

**How would you explain the main findings of your paper to non-scientific family and friends?**

Our lab studies ALS, a serious disease that affects nerve cells that control movement. People with ALS gradually lose the ability to move, speak and eventually breathe, and there is currently no cure. To understand the disease better, we used fruit flies that were genetically engineered to develop ALS-like symptoms. These flies gradually had more trouble walking and eventually became paralyzed and died earlier than normal, similar to the progression of ALS in patients. We found that the nervous system of these flies showed signs of ongoing inflammation, one of the body's natural defence responses to infections or injury. While this response is normally helpful, prolonged activation can damage healthy nerve cells. When we reduced this harmful response, the flies moved better and were able to walk more easily. Our findings suggest that excessive inflammation may contribute to ALS progression and that targeting it could help slow the disease in the future.

To better prepare young scientists for industry careers, greater exposure to industrial research during PhD training would be highly valuable

**What are the potential implications of these results for disease biology and the possible impact on patients?**

ALS remains one of the most difficult neurodegenerative diseases to treat. Despite being recognized for over a century, therapeutic progress has been limited, with only a handful of approved drugs that offer modest benefits. The complexity of the disease, driven by multiple genetic and cellular pathways, has made the identification of effective therapeutic targets particularly challenging. In our work, we identified Fos as a novel regulator of glial inflammatory responses in the *VAP^P58S^* model of ALS. Given the growing recognition that dysregulated neuroinflammation contributes to disease progression across a wide range of neurodegenerative disorders, understanding how Fos controls these pathways may reveal new opportunities for therapeutic intervention. Beyond ALS, these findings may have broader relevance to age-associated neurodegenerative conditions where chronic inflammation is a key pathological feature. With global demographics shifting toward an increasingly aging population and the population pyramid gradually inverting in many countries, the burden of neurodegenerative diseases is expected to rise substantially. Research that uncovers fundamental mechanisms linking inflammation, ageing and neurodegeneration may therefore have implications not only for disease treatment but also for promoting healthier ageing.

**Figure:**
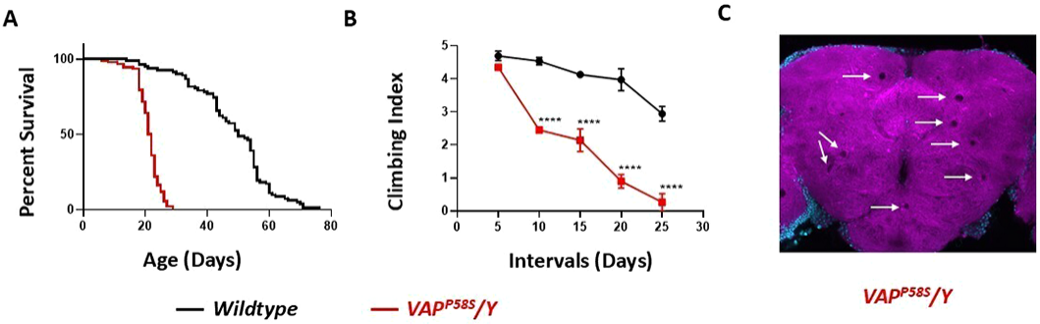
***VAP^P58S^* mutant flies show decreased lifespan, impaired motor function and brain vacuolation.** (A,B) Graphs showing percentage survival (A) and climbing index (B) in mutant and wild-type flies. The red lines depict the mutant and black lines depict the wild-type control. ****P<0.0001. (C) Representative image showing brain vacuolation in 20-day old mutant flies (indicated by arrows).

**Why did you choose DMM for your paper?**

DMM was a natural choice for our study because it focuses on diseases, disease models and the underlying mechanisms of disease progression, which are all central aspects of our work. The journal's emphasis on mechanistic insights in disease biology aligns closely with the questions we sought to address. I also appreciate DMM's commitment to open-access publishing, which ensures that our findings are accessible to the broader scientific community. Its strong focus on scientific rigor and research integrity further made it an ideal home for our work.

**Given your current role, what challenges do you face and what changes could improve the professional lives of other scientists in this role?**

I currently work as a Scientist – Skin Biology and Product Efficacy at Almora Botanica India Private Limited, where I lead the evaluation of skincare actives, design efficacy studies with Contract Research Organisation partners and manage cross-functional projects. I am also involved in setting up the scientific development pipeline for our company's new food supplement arm. One of the key challenges is building processes in a new and evolving area with limited existing expertise or infrastructure. Working in a startup environment requires a high degree of ownership, adaptability and independent problem solving. Another challenge is navigating the shift from the depth-focused approach of academia to the faster, outcome-driven pace of industry. Balancing scientific rigour with business timelines and practical constraints is an important part of the role. To better prepare young scientists for industry careers, greater exposure to industrial research during PhD training would be highly valuable. Structured collaborations, internships and industry-sponsored projects can help make this transition smoother. Additionally, formal training in project management and stakeholder engagement would equip early-career scientists with skills that are increasingly important in industry leadership roles.

**What's next for you?**

In the near term, I aim to establish robust scientific and testing pipelines for the food supplement portfolio at my organization. As this is a growing area, I am particularly interested in developing efficient processes, anticipating potential bottlenecks and building scalable frameworks that can support future product development. I see this as an excellent opportunity to learn, grow and strengthen my skills in translational product development and project leadership.

In the longer term, I would like to work on independent projects at the interface of academia and industry. My primary research interests lie in ageing and neurodegenerative disorders, and I hope to contribute to translational research that bridges mechanistic understanding of disease biology with the identification and development of novel therapeutic targets. Ultimately, I would like to play a role in translating fundamental scientific discoveries into meaningful interventions that can improve human health.

**Tell us something interesting about yourself that wouldn't be on your CV**

I love to travel and explore new places. PhD has been a fun ride on that front as it allowed me to travel a lot to present my work at both national and international conferences.
